# The Arming of Natural Killer Cells With Fc‐Engineered Monoclonal Antibodies Confers Specificity Against Tumor B Cells

**DOI:** 10.1002/mco2.70242

**Published:** 2025-07-04

**Authors:** Michaël Constantinides, Loïs Coënon, Paolo Falvo, Caroline Multrier, Davide Lombardi, Francesco Bertolini, Pierre Martineau, Bruno Robert, Guillaume Cartron, Martin Villalba

**Affiliations:** ^1^ IRMB, University of Montpellier INSERM CHU Montpellier Montpellier France; ^2^ Department of Clinical Hematology CHU Montpellier Montpellier France; ^3^ Laboratory of Hematology‐Oncology IRCCS European Institute of Oncology Milan Italy; ^4^ IRCM, University of Montpellier INSERM, ICM Montpellier France; ^5^ IRMB, University of Montpellier INSERM, CNRS, CHU de Montpellier Montpellier France

**Keywords:** ADCC, B‐cell chronic lymphocytic leukemia, engineered mAbs, NK cell therapy, non‐Hodgkin lymphoma

1

Dear Editor,

Monoclonal antibodies (mAbs) show clinical benefits in hematologic and solid cancers. Their efficacy relies on various mechanisms, including direct killing, complement‐dependent toxicity (CDC), antibody‐dependent phagocytosis (ADP), and antibody‐dependent cell‐mediated cytotoxicity (ADCC). Different modifications enhance mAb binding to Fc receptors (FcRs), including changes in the amino acid sequence [1]. Some modified antibodies are now approved or in clinical trials such as tafasitamab and margetuximab. However, effector cells are often dysfunctional in cancer patients [2], limiting the efficacy of mAbs and prompting the development of cell therapies.

Chimeric antigen receptors (CAR)‐T cells are approved by the FDA/EMA. Despite their substantial clinical success [3], they have some drawbacks, such as the necessity for autologous T cells to prevent graft versus host disease (GvHD). Moreover, their production is both challenging and expensive [3]. CAR natural killer (NK) cells could be an alternative to CAR‐T cells [3, 4], because NK cells do not induce GvHD and can consequently be employed in allogeneic settings [3, 4]. T and NK cells utilize degranulation of cytotoxic granules and engage death receptors through Fas and TRAIL. NK cells also mediate ADCC due to the expression of FcγRIIIa (CD16a) [5] and mAbs confer specificity to NK cells. Consequently, the use of allogeneic NK cells in combination with mAbs demonstrated promising results [5].

NK cells, in particular CAR NK cells [3, 4], are a promising alternative to address some drawbacks associated with CAR‐T cells. The clinical activity of allogeneic NK cells used as monotherapy is often insufficient [5]. Consequently, ongoing efforts are dedicated to enhance their activity and/or associate them to other therapies, notably by combining them with mAbs [3, 5]. We recently showed that SDH‐modified (S239D/H268F/S324T/I332E) mAbs can be loaded on NK cells, resulting in “armed” eNK cells with the mAb specificity (CAR‐like NK cells) [6].

Here we produced expanded and activated NK cells (eNK)[7] and demonstrated that SDH‐rituximab i) stays in the presence of human polyclonal IgG; ii) increases eNK killing against targets; iii) together with a SDH‐CD19, protects mice from CD20/CD19 heterogeneous human tumor xenograft. We first incubated RTX‐armed eNK in the presence of the polyclonal human IgG Privigen, which did not statistically decrease the percentage of SDH‐rituximab‐armed eNK or their MFI (Figure 1A), showing that an excess of human IgG did not replace SDH‐rituximab molecules on NK cell membrane.

**FIGURE 1:**
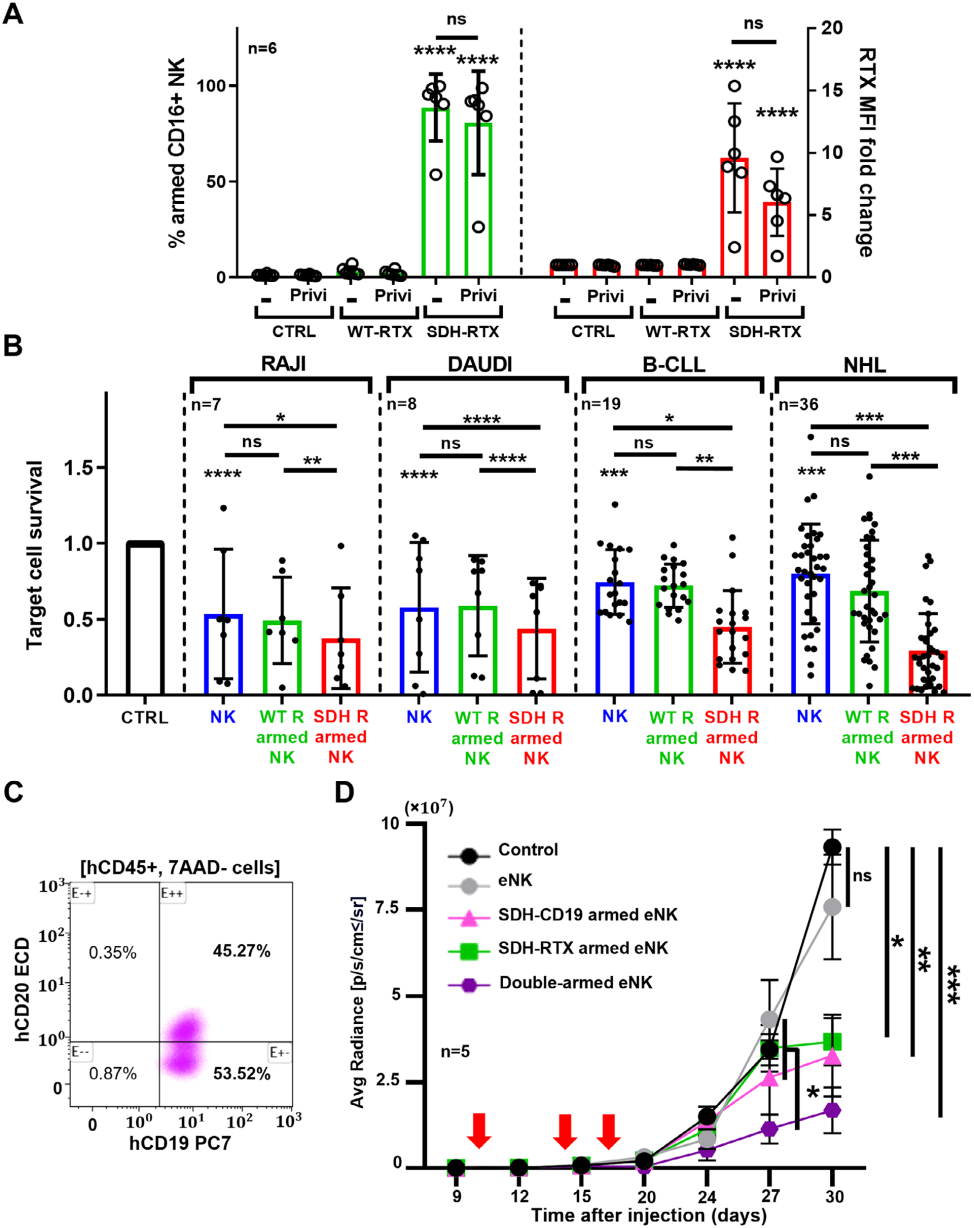
eNK arming stability in presence of human IgG and eNK cytotoxicity improvement in vitro and in xenograft model. A) percentage of eNK armed with RTX was, as well as the anti‐RTX MFI ratio normalized to non‐armed eNK 8 h after arming with 10 µg/mL of mAbs for 1 h, washing and incubation with 5 mg/mL of human polyclonal IgG (Privigen). The graphic represents the mean ± SD. B) Survival of target cell line target at E:T (1:1) with eNK (unarmed or armed with WT or SDH‐RTX) or primary tumoral cell at E:T (3:1), (10 B‐CLL with 9 eNK donors and 20 NHL with 16 eNK donors) after 8 h incubation. The graphic represents the mean ± SD of the survival ratio of RAJI or DAUDI cells (compared to MOLM‐13 cells) or tumor cell survival, normalized to 100%. C) Dot plot represents hCD19 and hCD20 expression by PDX cell line, selected as: 7AAD‐ and hCD45+ single cells. D) Depicts xenograft results for 25 male mice engrafted with the PDX line. Red arrows represent days (d10‐14‐17) of eNK injections (8 × 10^6^ cells). Graphics D represents mean ± SEM of average radiance reflection xenograft tumoral mass in the animals’ femurs. Tukey's test compared to control was used. *p < 0.05, **p < 0.01, ***p < 0.001, ****p < 0.0001.

Through cytotoxicity assay, eNK demonstrated natural killing on the CD20+ cell lines Raji and Daudi and SDH‐rituximab‐armed eNK showed improved cytotoxicity against non‐armed‐ and wt‐rituximab‐armed‐eNK (Figure 1B). We observed the same, but more accentuated, when we used primary tumor samples from B‐CLL and NHL patients in the leukemic phase (Figure 1B), demonstrating that SDH‐rituximab‐armed eNK exhibited improved cytotoxicity on CD20+ cells

Next, we engrafted mice with a patient‐derived xenograft (PDX) [8]. All tumor cells express CD19, but only a half express CD20 (Figure 1C), allowing us to investigate the comparative effect of single‐armed and double‐armed eNK. We developed an anti‐CD19‐SDH mAb based on the blinatumomab (SDH‐BLI) and single‐ or double‐armed eNK with this and SDH‐rituximab. Both single‐armed eNK improved mice survival compared to non‐armed‐eNK (Figure 1D). Double‐armed eNK cells were more active than all other conditions (Figure 1D).

We demonstrated here that eNK arming using SDH–rituximab withstands competition with physiological concentrations of human polyclonal IgG, indicating that armed eNK may retain mAb when administered to patients. Direct loading of the mAb onto the effector should offer potential clinical advantages: (i) circumventing undesirable side effects of mAbs, such as ROS production by neutrophils following antibody binding to their FcR, which inhibits NK cell‐mediated ADCC; (ii) reducing the amount of mAb, thereby decreasing the risk of immunization to the antibody; (iii) loading multiple mAbs in the same effector cells could mitigate the risk of tumor escape through the downregulation of a single antigen. The last is supported by our results (Figure 1D).

Cells were collected, prepared and stored as previously described [7]. After thawing, patient's PBMCs recovered for 4–8 h in RPMI1640 glutamax+10%FBS (Thermo Fisher Scientific). NK cell isolation and expansion were performed from UCB [6, 7]. We used the PLH Epstein–Barr Virus (EBV) transformed lymphoblastoid cell line to produce eNK as described [7]. Raji and Daudi were obtained from ATCC. The MOLM‐13 (AML‐M5a) cell line was obtained from Dr. J.E. Sarry (INSEMU1037, INSERM, France). Cells were cultured in RPMI1640 glutamax+10%FBS. The luciferase‐expressing patient‐derived xenograft (PDX) line DFBL‐69487‐V3‐mCLP was obtained from the Public Repository of Xenografts (www.proxe.org).

Privigen, a human polyvalent IgG mix, and RTX were obtained from the University Hospital of Montpellier. S239D/H268F/S324T/I332E modified RTX (SDH‐RTX) was produced in CHO cells and purified by protein A (Evitria, Zürich, Switzerland or RD‐Biotech, Besançon, France). These mutations have been previously described [1, 5]. SDH‐anti‐CD19 mAb was based on the anti‐CD19 scFv sequence from the blinatumomab (CD3×CD19 bispecific T cell engager) [9] and produced by RD‐Biotech.

eNK were armed with 10 µg/mL of mAbs (WT‐RTX, SDH‐RTX) for 1 h before washing [6] and incubated for 8 h (37°C, 5% CO_2_) in medium with or without 5 mg/mL of Privigen before staining. eNK arming was revealed by an anti‐RTX idiotype recognizing both WT‐RTX and SDH‐RTX (Method 1 in the Supporting Information “Competition staining mix”) and analyzed as in Method 2 in the Supporting Information.

For the cytotoxicity assay, cells lines (1.4 × 10^6^ cells/mL) were stained with CellTrace Far Red (CTFR), 1:2500° for target cells, and CellTrace CFSE (CTG) (both Thermo Fisher scientific France), 1:5000° for control MOLM‐13 cells. We placed 2 × 10^5^ of each target and control cells and eNK in a 96 wells U bottom plate and incubated them for 8 h (37°C, 5% CO_2_). Cells were stained using 20 µL of 1:200 Viobility 405/452 and eNK were stained with an anti‐CD56 (Miltenyi Biotec, clone: REA 196). Survival ratio was calculated by comparing numbers of surviving MOLM‐13 and target cells [10].

Patient's PBMCs (2 × 10^5^ cells) were seeded in 96U well plates and CTFR (1/2500°) stained eNK were added at an effector/PBMC ratio of 3:1. After 8 h, cells were washed and stained for cell death (Viobility 405/520) and “tumor B‐cells assessment staining mix” (Supporting Information S1). We added precision counting beads (20 µL) and analyzed as in Method 3 in Supporting Information.

Mice for the xenograft experiments were bred and housed at the Institute of Molecular Oncology (IEO–IFOM, Milan, Italy). 10^6^ PDX DFBL‐69487‐V3‐mCLP cells were injected in the tail vein of 8‐week‐old male NSG mice. Tumor growth was monitored three times per week by whole‐body imaging on an IVIS Lumina III platform. After 10 days, tumors were quantified and mice randomized in five experimental groups each containing five mice: control, eNK, RTX‐armed eNK, CD19‐armed eNK and double (RTX+CD19)‐armed eNK. An 8 × 10^6^ of eNK were resuspended in 200 µL of PBS supplemented with 100IU/mL rhIL‐2 (Thermo Fisher Scientific, France) and 5 ng/mL hrIL‐15 (Miltenyi Biotec, Bergisch Glabach, Germany) and injected via tail vein at Days 10, 14, and 17. Xenograft mass radiance was evaluated as previously described [8].

Cytometry experiments were conducted using a Gallios 3 Lasers (Beckman Coulter, Pasadena, CA, USA) or a Symphony A3 5 lasers (BD biosciences, France) flow cytometers. Acquisition was carried out using Kaluza software V1.3 (Beckman Coulter) or Facs Diva (BD biosciences France).

Statistics were performed using Prism V7.04 software. Each sample value represents the average of, at least, a technical duplicate.

## Author Contributions

M.V. and G.C. supervised the project. M.V., G.C., and F.B. obtained funding to realize the research project. M.V., G.C., M.C., P.F., F.B., and L.C. designed experiments. M.C., L.C., C.M., collected samples and performed in vitro experiments. P.F. and D.L. performed all in vivo experiments. M.C. and L.C. collected data, analyzed data and performed statistical analysis. M.C. and M.V. interpreted data. M.C. and M.V. wrote the manuscript. G.C. gave access to patient's samples. B.R. and P.M. designed and produced modified antibodies. All authors have read and agreed with the manuscript.

## Ethics Statement

Patients were enrolled in the HEMODIAG_2020 cohort (ID‐RCB: 2013‐A00260‐45, NCT02134574, CHU Montpellier) and provided written informed consent. Umbilical cord blood units (UCBs) were sourced from the Biological Resource Center Collection of the University Hospital of Montpellier—(BIOBANQUES Identifier—BB‐0033‐00031, CHU Montpellier).

Experiments involving animals were approved by the Italian Ministry of Health and have been performed in accordance with the applicable Italian laws (D.L.vo 26/14 and following amendments), with the Institutional Animal Care and Use Committee and with the institutional guidelines of the European Institute of Oncology.

## Conflicts of Interest

The patent WO2022023581A1 “Armed NK cells for universal cell therapy” have been filed by M.V., P.M., and B.R. and is licensed to CYTEA BIO. M.V., P.M., and B.R. have been remunerated as advisors by the company CYTEA BIO. The authors declare no other conflicts of interest.

## Supporting information




**Supporting File 1**: mco270242‐sup‐0001‐SuppMat.pdf.

## Data Availability

Data are available on demand to the corresponding author.
