# Supplementary material for: The Arming of Natural Killer Cells With Fc‐Engineered Monoclonal Antibodies Confers Specificity Against Tumor B Cells
Source: MedComm (2020). 2025 Jul 4;6(7):e70242. doi: 10.1002/mco2.70242 (PMC12231191; doi:10.1002/mco2.70242)
Supplement: Supplementary file 1 — Supporting File 1: mco270242‐sup‐0001‐SuppMat.pdf. [file MCO2-6-e70242-s001.pdf]

Competition staining mix

Parameter	Provider	Clone	Catalog number	Dilution/Concentration
Cell death	Thermo-fisher sci	EF780	65-0865-14	1:1000°
CD16	Miltenyi	REA423	130-113-393	1:200°
CD56	Miltenyi	REA196	130-114-551	1:200°
Rituximab idiotype	R&D system/ biotechne	2260A (rabbit)	CSA3135	1:50°

Tumor B-cells assessment staining mix

Parameter	Provider	Clone	Catalog number	Dilution/Concentration
CD19	Miltenyi	REA675	130-113-645	1:200°
CD20	BD biosciences	L27	347201	1:200°
CD10	BD biosciences	MEM-78	750190	1:200°
CD5	BD biosciences	UCHT2	612842	1:200°
CD23	BD biosciences	EBUC-5	743430	1:200°
CD79a	Miltenyi	HM47	130-104-226	1:200°
K light chain	BD biosciences	G-20-193	743168	1:200°
λ light chain	BD biosciences	JDC-12	748804	1:200°

Supplemental method 1. cytometry panels used in the study

Tables show analysed parameters, providers, clones, provider's catalog numbers and concentration (for chemical) or dilution (for mAbs) used to obtain all results presented in the article. Base for staining mix is PBS +2% FBS, (+10% brilliant stain buffer (BD biosciences) for experiment relative to patient's primary cells). Final staining volume is 100µL in all cases. Incubation time was followed by at least 2 washing steps using PBS+2% FBS. Staining mix were used to stain until 600 000 viable cells per sample.

A

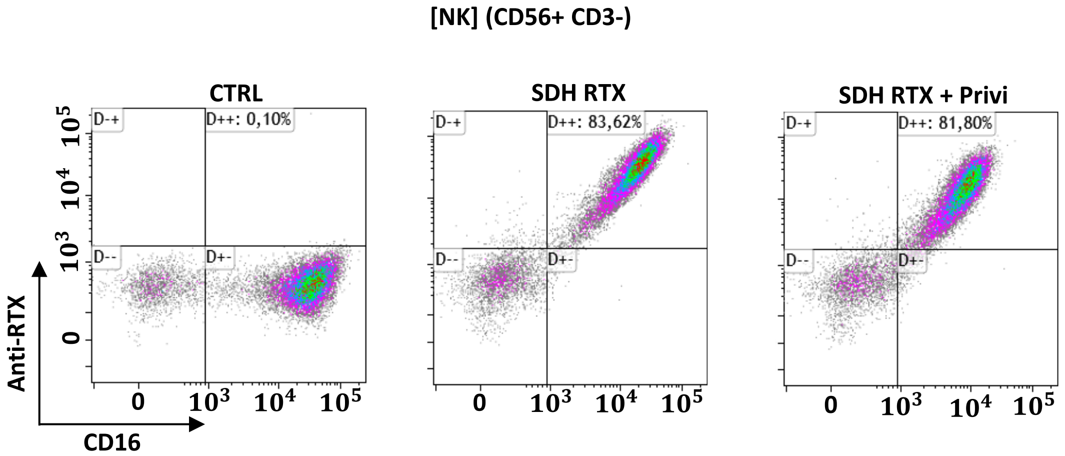

Supplemental method 2: eNK arming in presence of human polyclonal IgG, i.e. Privigen®. eNK were armed for 1 h with 10 µg/ml of the depicted mAbs, washed and incubated with 5 mg/mL of human polyclonal IgG (Privigen®) for 8 h. The percentage of eNK armed with RTX was quantified with an anti-RTX idiotype antibody. The MFI ratio was normalized to non-armed NK cells.

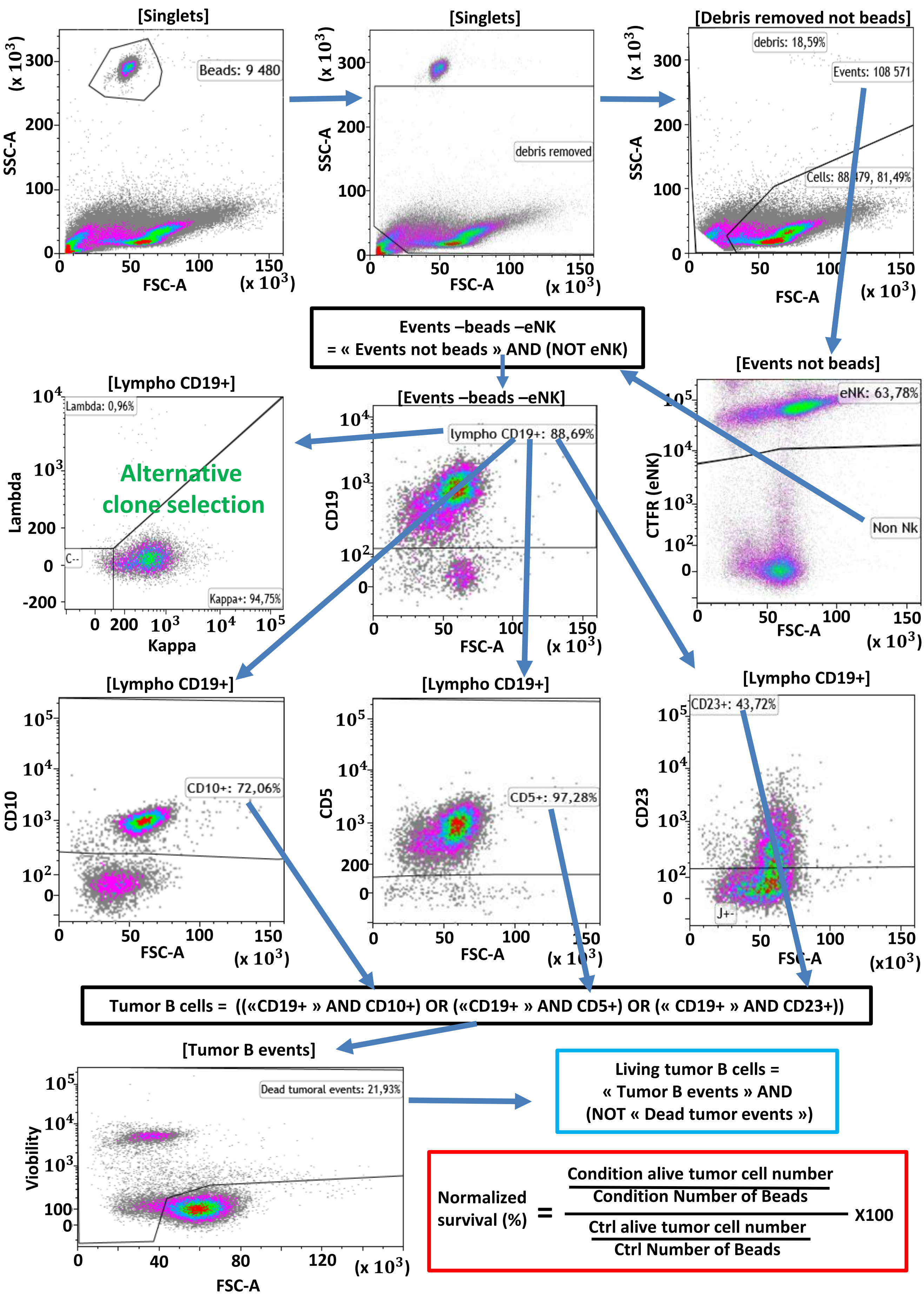

Supplemental method 3. Gating strategy used to select tumor B cells and to assess their viability after co-culture with eNK. Doublets were removed using FSC-H/FSC-A dot plot prior to this analysis. We used a BD symphony A3 cytometer and BD facs diva software to acquire data. All data were analyzed using the Beckman Coulter Kaluza Analysis 2.1 software.
